# Department of the Arts and Sciences

**Published:** 1869-10

**Authors:** 


### Department of the Arts and Sciences.

The scientific columns of this Journal will hereafter be devoted to a careful selection of matter relating to new discoveries and inventions, and will be open to the review of subjects which may prove interesting to every class of readers. Persons desiring information respecting patents, the Patent Office at Washington, novelty and patentability of new inventions, as also the procuring for them of patents in the United States and foreign countries—may consult this office.

Parties having inventions, without the knowledge or mechanical skill necessary to reduce them to practical utility, may have them perfected. Patents may be secured without the invention becoming public.

Illustrations of new and valuable inventions we are prepared to give, executed by the best artists, and noticed in the Journal when requested.

Address Patent Department of

Hall's Journal of Health, Art and Science,
176 Broadway, New York.

Since the first year of this Journal, the Editor has written all the articles which have appeared in its pages, except in a very few instances where the words of others were given in illustration of positions taken. Usually twenty pages were given in each number pertaining to health and disease; it is proposed to add eight pages of matter, original and selected, on subjects pertaining to human progress in general, so as to have a wider range and afford instructive and practical reading for all classes:

The Farmer. The Bar.
The Mechanic. The Bench.
The Artisan. The Pulpit.

Something which may be profitably read in every household.

The aim will be to present established facts in art and science as a means of promoting the intelligence of the people, their elevation and their general well-being.

Invention and Discovery.—The world can never repay its obligations to those patient experimenters and hard thinkers who have added so much to the comfort of civilized society, and whereby their discoveries have saved time and labor and expense to an incalculable amount. Hinckley's knitting machine performs as much work in one day as ten women One sewing machine will do in an hour what one person formerly required a dozen to accomplish. One of McCormick's reapers does the work of a dozen men.

The steam power brought into practical use by Fulton and Watt performs in the United States the work of a hundred and thirty millions of men, and in Great Britain that of four hundred millions more ; it carries a man in one day the distance of ten. Within easy memory, horseback travellers thought they were doing well on long journeys to average thirty or forty miles a day ; now, a man can sleep and travel and make at the rate of three or four hundred miles of travel in twenty-four hours. Statements of similar facts might be indefinitely extended, but these show how much the world is indebted to inventors, and to the printing press for its speedy distribution of the knowledge of these things among the people, so that they may make a practical use of them, and thereby be benefited by them. It is with this view that it is proposed in future to give eight pages of additional reading matter to our subscribers pertaining to invention, improvement and discovery in all departments of human life, and thus make it a welcome visitor to every household in the United States.

